# Metastatic Adrenocortical Carcinoma With Chromothripsis: A Case Report and Literature Review

**DOI:** 10.7759/cureus.41218

**Published:** 2023-06-30

**Authors:** Gyuhee Seong, Alexander Wu, Charles Kim, Nirmal Pathak, Elif Yakut, Zhonghua Li, Edwin Chiu

**Affiliations:** 1 Department of Medicine, State University of New York (SUNY) Downstate Health Sciences University/Kings County Hospital Center, New York, USA; 2 Department of Hematology and Oncology, State University of New York (SUNY) Downstate Health Sciences University/Kings County Hospital Center, New York, USA; 3 Department of Pathology, State University of New York (SUNY) Downstate Health Sciences University/Kings County Hospital Center, New York, USA

**Keywords:** mitotane, micronuclei, comparative genomic hybridization, genome instability, myxoid, chromothripsis, metastatic adrenocortical carcinoma

## Abstract

Metastatic adrenocortical carcinoma (ACC) often has a poor outcome, with a five-year survival of less than 25%. We report a rare case of metastatic ACC with a myxoid variant with chromothripsis. We review the histologic variants of ACC, including myxoid type, molecular drivers, and current and investigational therapies for adrenocortical carcinoma. We also discuss the mechanism of chromothripsis, chromothripsis in ACC tumorigenesis, and propose potential therapies targeting chromothripsis.

## Introduction

Adrenocortical carcinoma (ACC) is a rare disease with an incidence of approximately 0.7 to 2 per million population annually [1]. It often has poor outcomes, with a five-year survival of 60-80% for tumors localized in the adrenal gland, 35-50% for locally advanced ACC, and 0-28% for metastatic ACC [2]. At the time of diagnosis, most patients with ACC have either locally advanced disease (34% with stage III) or metastatic disease (26% with stage IV) [1], often with hormonal hypersecretion that increases morbidity [3].

Complete surgical resection is the only known curative therapy for ACC [3]. Mitotane is a derivative of the insecticide dichlorodiphenyltrichloroethane (DDT). It is a steroidogenesis inhibitor and adrenolytic drug used as adjuvant therapy for patients with ACC after complete surgical resection who have either stage III disease, R1 resection, or high-grade disease of any stage (Ki67 index >10%).

Chromothripsis is a single catastrophic genome reshuffling that generates up to thousands of genomic rearrangements in one or a few chromosome(s) [4]. In 2011, Stephens et al. first described chromothripsis when massive genome rearrangements were detected in patients with chronic lymphocytic leukemia. “Thripsis” in Greek means “shattering” into small fragments [5].

Chromothripsis is now generally accepted as a widespread mutational phenomenon [6]. Chromothripsis challenges the traditional paradigm of the mutational accumulation theory of oncogenesis. Chromothripsis is an “explosive” mechanism of rapid destabilization of the cellular genome in nature, and this genome instability plays a prominent role in tumor onset as an early event in tumor development [4,6]. Whole-genome sequencing revealed that chromothripsis affects a substantial proportion in 28 tumor types and prevalence was 49% across all cases [4].

Here we report a rare case of metastatic adrenocortical carcinoma with chromothripsis.

## Case presentation

A 74-year-old woman from Saint Kitts with a medical history of functional dyspepsia presented to the hospital for five months of progressively worsening epigastric pain, bloating, early satiety, weight loss, and generalized weakness. The patient had a computerized tomography (CT) of the abdomen in her home country, which reported an interval increase of adrenal mass from 5.1 x 4.0 cm to 9.7 x 7.2 cm compared to three years ago. The patient never smoked cigarettes or used alcohol.

Vital signs were significant for elevated blood pressure. Physical exam was remarkable for moon faces, bilateral pitting lower extremity edema from the ankles up to shins, and diffused ecchymosis in upper and lower extremities. Labs were significant for hypokalemia (2.7 mmol/L), hyperglycemia (glucose 300 mg/dL), high cortisol (33.97 μg/dL, not suppressed by 1 mg dexamethasone test), and suppressed adrenocorticotropic hormone (ACTH) <5 pg/mL, consistent with Cushing syndrome, aldosterone 3.1 ng/dL, and plasma renin activity 0.7 ng/ml/hour. Plasma metanephrine 18 pg/ml, plasma normetanephrine 54 pg/ml, and urine metanephrine 60 mcg/24h were all within normal range, which ruled out pheochromocytoma. CT abdomen and pelvis with contrast showed a 6.7 x 7.8 x 11cm left adrenal mass. Magnetic Resonance Imaging (MRI) of the abdomen with contrast and CT chest did not show any evidence of metastasis.

**Figure 1:**
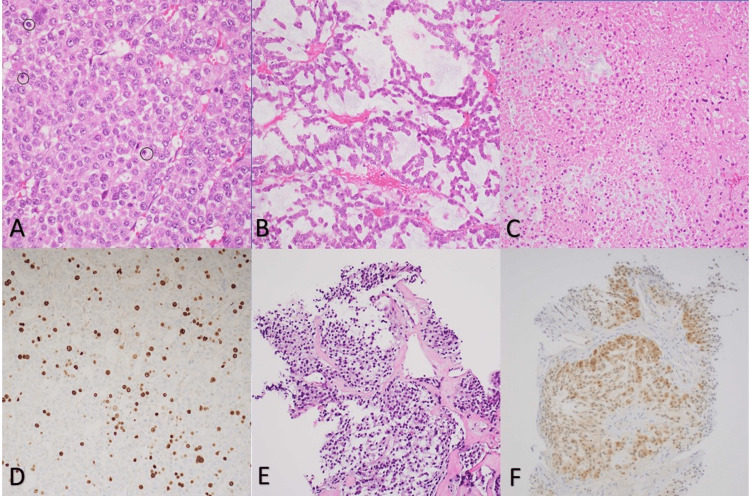
Pathology slide of left adrenal gland (A-D) and lung (E, F) Left adrenalectomy shows adrenocortical neoplasm composed of diffuse neoplastic epithelial cell proliferation with brisk atypical mitosis (A. H&E, x 200, atypical mitotic figures circled), focal myxoid features (B. H&E, x 200), focal necrosis (C. H&E, 200x), proliferative index (Ki-67) focally up to 15% (D. Immunohistochemistry, x 200), capsular and lymphovascular invasions (data not shown). Three years later, a lung core needle biopsy of one pulmonary nodule revealed a tumor with similar morphology to the adrenocortical neoplasm (E. H&E, x 200) and immunoreactivity with calretinin (F. Immunohistochemistry, x200) and melan A (data not shown), consistent metastatic adrenocortical carcinoma.

The patient underwent left adrenalectomy which showed a large adrenocortical neoplasm with focal myxoid features (less than 10% of the tumor), a proliferative index (Ki-67) focally up to 15%, and Weiss score 7 - Diffuse architecture, necrosis, capsular invasion, lymphovascular invasion, clear or vacuolated cells <=25% tumor, and atypical mitotic figures 25/50 high power fields (>5/50 HPF) (Figure 1A-1D). Wild-type p53 expression was detected by immunohistochemistry.

The patient was started on oral hydrocortisone and later received adjuvant radiation (180cGy x 28 fractions, total 5040cGy dose to the postoperative bed) due to high-risk features (high Ki67 mitotic index). The patient was started on adjuvant mitotane. However, she did not adhere to mitotane therapy as she experienced side effects such as vertigo, severe fatigue, diarrhea, headaches, and blurry vision.

Repeat CT chest and abdomen/pelvis with contrast at 16 months after initial diagnosis reported interval development of new, scattered bilateral pulmonary nodules, with the largest measuring 0.9 cm. Unfortunately, the patient was lost to follow-up during the coronavirus disease 2019 (COVID-19) pandemic.

She returned to the oncology clinic two years later. CT chest, abdomen and pelvis with contrast at 30 months after initial diagnosis showed interval progression of innumerable pulmonary nodules, a new right breast 1 cm lesion, a new lesion 1.5 x 1.2 x 2.7 cm in the small bowel, 2.4 x 1.7 cm round lesion along the serosa of the small bowel, 1 cm round lesion in the cecum, and thickening of the gastric wall to 2.1 cm predominantly of the greater curvature along the fundus and body. Core needle biopsy of one pulmonary nodule by Interventional Radiology revealed metastatic adrenocortical carcinoma confirmed by immunohistochemistry - carcinoma positive for cytokeratins AE1/AE3, calretinin, inhibin, melan-A, and synaptophysin (Figure 1E, 1F).

Diagnostic mammogram and ultrasound of the 2.4 cm irregular hypoechoic right breast mass, previously measuring 1.4 cm, was consistent with invasive ductal carcinoma (grade 2, ER+/PR+/Her 2 by IHC with score 1+). The case was presented in a multidisciplinary breast conference, and the consensus was to treat her breast cancer with primary endocrine therapy with anastrozole and CDK4/6 inhibitor (palbociclib) instead of surgery, considering the patient's prognosis from her metastatic adrenal carcinoma.

The patient was referred to genetic counseling and was found to have a gross deletion of chromosome 11 and identified mutations in the MRE11A (11q21), SDHD (11q23), and LZTR1 (22q11.2) genes on genetic testing. Further high-resolution chromosomal microarray analysis reported chromothripsis, gross deletions involving the short and long arms of chromosomes 2 and 11 and the short arm of chromosome 16. Flow cytometry and peripheral blood smear did not suggest any evidence of hematologic malignancy.

It has been five years and four months (64 months) since the patient was diagnosed with Stage II ACC, and one year and 11 months (23 months) since the diagnosis of metastatic ACC. The patient currently remains on mitotane therapy.

## Discussion

Epidemiology, etiology and prognosis of ACC

Adrenocortical carcinoma (ACC) is a malignancy in the adrenal cortex. The distribution of ACC is bimodal. The first peak of ACC occurs in early childhood (age five and under), while the second peak occurs in adults (age 40 to 50) [7].

Although most of ACC's etiology is either sporadic or unknown, a few cases are occasionally associated with other syndromes. These syndromes include Li-Fraumeni syndrome (TP53 gene germline and somatic mutation), Lynch syndrome (MSH2, MLH1, MSH6, PMS2, EPCAM genes), multiple endocrine neoplasia type 1 (MEN1 gene), Beckwith-Wiedemann syndrome (11p151 gene, IGF-2 overexpression), familial adenomatous polyposis (FAP gene, β catenin somatic mutations), neurofibromatosis type 1 (NF1 gene) and Carney complex (PRKAR1A gene) [8].

Median survival time was 24.1, 6.08, 3.47, and 0.89 years for stages I, II, III, and IV ACC, respectively, in a retrospective review of 330 ACC patients in 2015 by Ayala-Ramirez et al. Despite the generally poor prognosis of ACC, there is a significant individual variance in overall survival. Even among stage 4 ACC cases, the survival rate varies from a few months to many years. The clinical course of approximately 5% of all ACC patients in the Michigan Endocrine Oncology Repository will extend longer than 10 years [9]. Investigations on the prognostic indicators for longer survival are underway. Our patient has lived longer than 64 months since diagnosed with ACC and 23 months since the diagnosis of metastatic ACC, survived longer than median expectancy.

Histology of ACC and myxoid variant 

The Weiss criteria on hematoxylin-eosin stained slides are the most used histopathologic tool for distinguishing between an adrenocortical adenoma and an ACC.

Three or more Weiss criteria strongly indicate ACC: high nuclear grade, more than five mitoses per 50 high-power fields, atypical mitotic figures, less than 25% clear cells, diffuse architecture, necrosis, venous invasion, sinusoidal invasion, and capsular invasion [3].

There are three histologic variations of ACC in addition to the conventional ACC: oncocytic, myxoid, and sarcomatoid. A myxoid variant is an uncommon variant of ACC that accounts for 10% of all ACC cases [10,11]. It is characterized by the variably abundant extracellular myxoid components (5-90%) resembling carcinomas with extracellular mucin secretions or myxoid tumors. Myxoid variants have two distinct morphologic groups [10]. Group 1 is a predominantly myxoid stromal component with pools of copious mucin, and group 2 is focal myxoid changes (less than 20%) in tumors otherwise similar to conventional ACC. Group 1 may represent true myxoid ACC as a distinctive subgroup, whereas group 2 may be the result of myxoid degenerative changes of conventional ACC.

In a retrospective study in a single center, those with myxoid or sarcomatoid ACC had a median survival of 0.7 years compared to 1.9 years in individuals with conventional ACC and had clinical manifestations that were slightly more aggressive [12]. The patient of this case has ACC with focal myxoid changes of less than 10% and has a long survival.

Current treatment of ACC

ACC is commonly diagnosed during the evaluation of endocrinologic conditions such as hypercortisolism, Cushing syndrome, hyperandrogenism, primary aldosteronism, or from an incidental finding on imaging [3]. Due to its rarity and short survival, there are few randomized trials for treatment of ACC. Thus, the treatment of ACC is based on consensus opinion and lower grades of evidence.

TNM staging American Joint Committee on Cancer (AJCC) 8th edition and European Network for the Study of Adrenal Tumours (ENSAT) staging are widely used for classification [2].

The gold standard for treatment of ACC with curative intent is open surgery [3]. Laparoscopic resection is associated with a higher risk of recurrence, tumor rupture, and carcinomatosis [1]. Based on a randomized ADIUVO study, patients with a low-intermediate risk of recurrence are usually observed following curative surgery [13].

Mitotane utilized as adjuvant therapy for locoregional/stage 3 ACC with high-risk features is based on evidence from retrospective observational studies [14]. Adjuvant mitotane therapy is generally continued for at least two years unless patients cannot tolerate it or have significant recurrence despite optimal mitotane dosage. Mitotane levels should be measured and used to monitor the effect of therapy. The therapeutic mitotane level of the target is between 14 and 20 mg/L [1].

In metastatic ACC, mitotane is the mainstay of treatment. When there are isolated, resectable lesions, surgery followed by mitotane therapy is recommended. Based on phase III FIRM-ACT trials, platinum-based chemotherapy such as etoposide, doxorubicin, and cisplatin with mitotane (EDP-M) can be considered in the metastatic setting. Radiation is recommended for those with a high risk of recurrences, such as stage III disease, R1 resection, and high-grade or intraoperative or capsular rupture [3].

An ongoing phase III randomized trial (ADIUVO-2) compares adjuvant mitotane alone to the mitotane with three months of chemotherapy (cisplatin and etoposide) in high-risk ACC after primary surgical resection [1].

Molecular target and current trials of ACC 

The most common mutations in sporadic ACC include insulin-like growth factor 2 (IGF2), β-catenin (CTNNB1 or ZNRF3), and TP53 mutations [8].

IGF-2 overexpression was observed in more than 85% of ACCs, despite being low or absent at the start of clonal expansion. IGF2 activates tyrosine kinase receptors that trigger mitogen-activated protein kinase (MAPK) and phosphatidylinositol 3-kinase (PI3K)/Akt pathway and subsequent mTOR pathway. The canonical Wnt/β-catenin pathway, especially the β-catenin protein, is crucial in ACC tumorigenesis. Tumor protein 53 (TP53, p53) is a protein of the tumor suppressor gene. Twenty percent to 30% of sporadic ACC had somatic TP53 mutations, associated with poor outcomes [8].

Potentially targetable mutations in ACC were identified in next-generation sequencing (NGS) of 107 patients, including CDK4, notch signaling, NF1 in RAS/MAPK, MDM2 in the p53 pathway, BRACA1/2, ATM, BRAF, PTCH1 as sonic hedgehog receptor, mTOR pathway, EGFR/KIT/RET as receptor tyrosine kinase, estrogen receptor, and EZH2 (histone N-methyl-transferase) [15].

Targeting insulin-like growth factor receptors, tyrosine kinase receptors, or mTOR has not been effective to date [16].

Activation of the cMET pathway (receptor tyrosine kinase) is one of the reported mechanisms of resistance to therapy in ACC after exposure to commonly used therapies (cisplatin, radiation, and mitotane). Targeting the cMET pathway in ACC could be promising. There are three ongoing trials to assess the safety and efficacy of cabozantinib, a small molecule inhibitor of c-Met and VEGFR2, AXL, and RET [1].

There are clinical trials with immune checkpoint inhibitors - combined therapy with nivolumab and ipilimumab (NCT02834013 and NCT03333616) and one with pembrolizumab (NCT02721732). 

Chromothripsis

Chromothripsis, an extensive single genomic reshuffling, leads to high a frequency of deletions with an almost complete absence of duplications in localized genomic regions [17].

Mechanism of Chromothripsis

Chromothripsis is caused by exogenous and endogenous factors which trigger chromosome shattering and sequential reassembly of fragments through micronuclei formation, telomere shortening through breakage-fusion-bridge cycles in dicentric chromosomes, aberrant epigenetic regulation, abortive apoptosis, and other unknown mechanisms [17].

Although these mechanisms are not mutually exclusive, the most supported mechanism for chromothripsis is chromosome damage and missegregation within micronuclei. Micronuclei are small, round, extra-nuclear structures consisting of a lipid bilayer surrounding DNA [18].

Chromothripsis and Cancer

Chromothripsis was initially identified in a patient with chronic lymphocytic leukemia [5]. Chromothripsis is likely an early event that drives tumorigenesis [18]. It is a primary mechanism accelerating DNA amplification and acquisition of tolerance to modified growth conditions in tumorigenesis [19]. The incidence of chromothriptic rearrangements in cancer is significantly higher than previously assumed and could reach 100% in some types of cancer [6].

Chromothripsis is not limited to cancer but has also been reported in the germline, predominantly in patients with dysmorphic features or developmental delay [18].

Prognosis of Chromothripsis in Cancer

Various literature has reported that chromothripsis is more common in aggressive cancer and is associated with shorter survival. Rapid accumulation of multidrug resistance or the localization of many oncogenes in double minutes could explain this [6]. For instance, chromothripsis was significantly associated with shorter overall survival in metastatic colorectal cancer (n=33) [4].

Although it has not been verified in ACC yet, patients with chromothriptic tumors could benefit from aggressive treatment, given the strong association between chromothripsis and poor prognosis in several tumors. Chromothripsis scoring may help guide patient stratification [20].

Genomic drivers and chromothripsis in ACC

Potential driver genes in sporadic ACC are proposed, such as insulin-like growth factor 2 (IGF2, 11p15.5), β-catenin (CTNNB1, 3p22.1), TP53 (17p13.1), ZNRF3 (22q12.1) and TERT (5p15.33), as well as novel drivers such as PRKAR1A (17q24.2), RPL22 (1p36.31), TERF2 (16q22.1), CCNE1 (19q12) and NF1 (17q11. 2) and deletions of RB1 (13q14.2), CDKN2A (9p21.2), and CDK4 (12q14.1) [8,21].

Chromothripsis in ACC (n=15) is predominantly reported in chromosomes 17, 19, and 22, affecting driver genes that have an essential role in ACC, such as PRKAR1A (17q24.2), MLL4 (19q13.12), CCNE1 (19q12) and ZNRF3 (22q12.1) [4].

Interestingly, chromosome regions frequently gained or lost in tumors with chromothripsis were also frequently gained or lost in those without chromothripsis. This suggests that different processes other than chromothripsis alter the copy numbers in a non-random fashion and grant selective advantages to the affected cells [4].

The patient in our case had chromothripsis in the short and long arms of chromosomes 2 and 11 and the short arm of chromosome 16 on comparative genomic hybridization (CGH). Genetic mutations in MRE11A (11q21) and SDHD (11q23) genes and LZTR1 gene (22q11.2) were detected as well. In a chromothripsis frequency analysis per chromosome of ACC, although chromosomes 17, 19, and 22 were affected the most, chromosomes 2 and 11 as in this case were affected by chromothripsis [4]. This patient in this case had MRE11A (11q21), and SDHD (11q23) genes and LZTR1 gene (22q11.2) mutations, and they are often mutated in cancers (1.30%, 0.45%, 0.53% respectively) [22]. In regard to relationship with ACC specifically, there is no proven relationship with MRE11A gene mutation. There are reported case series for SDHD mutation in four ACC patients (Else et al., 2017) [9]. High expression of LZTR1 is recently proven to be strongly associated with worse outcomes in ACC (Zhou et al., 2022) [22].

We adopted the comparative genomic hybridization (CGH or microchip chromosome analysis) method to detect chromothripsis, which allows for detecting abnormalities in the copy number of genes, such as loss and gain in DNA. Due to the limitation of CGH, we could have underestimated the prevalence of chromothripsis in this patient. CGH cannot detect balanced structural or minimal chromosomal aberrations or determine the order and orientation of derived chromosomal segments. NGS would more accurately determine the localization of breakpoints and nucleic acid sequences at breakpoint junctions in the whole genome, despite the high cost and difficulty of detecting large translocations and deletions [6].

Potential therapeutics targeting chromothripsis

We reviewed potential therapeutic strategies targeting chromothripsis in cancer. First, a strong link exists between homologous recombination deficiency and chromothripsis in human tumors and genetic mouse models. Therefore, like PARP inhibitors in homologous recombination deficient breast and ovarian cancer, PARP inhibitors could be utilized in tumors with chromothripsis [20].

Second, DNA damage response and mitotic defects in chromothriptic tumors could be further investigated. Targeting residual DNA damage response pathways could effectively treat chromothriptic tumors lacking DNA damage response [20].

Third, chromothriptic tumors have five times more fusion genes than other structural variants. As several fusion genes offer druggable targets and diagnostic markers, this will benefit the search for druggable targets in tumors with chromothripsis [4]. As chromothripsis is currently viewed as an early event in tumorigenesis, such druggable genes may be of particular therapeutic value by offering clonal targets [20].

Fourth, chromothriptic tumors may display higher sensitivity to immune therapies due to many genomic rearrangements. Chromosome missegregation and consequential micronuclei formation provide a confined environment for chromothriptic events. They can also be considered as triggers of the innate immune response by activating cGAS-STING signaling. STING is an essential component in the recruitment of immune cells to the tumor microenvironment and in the immunologic clearance of the tumor. Therapeutic activation of cGAS-STING signaling might provide more pronounced immune response against tumor cells with chromothripsis [18]. The role of immunotherapy in ACC is not established yet, which could be explained due to low PD-L1 expression, or diminished CD8+ production due to the presence of TP53 mutations and WNT-B-catenin [16].

All these therapies mentioned above targeting chromothriptic tumors are investigational, and their application to adrenocortical carcinoma treatment requires further research.

## Conclusions

We report a rare case of metastatic adrenocortical carcinoma (ACC) with a myxoid variant of less than 10% with chromothripsis. Considering the poor prognosis and limited curative options in ACC, this case exemplifies the importance of understanding the mechanism of chromothripsis, and further research on the potential therapeutic application in ACC is warranted.
